# Nursing Home Work Environment and Burnout Association in Registered Nurses and Nursing Assistants: A Cross‐Sectional Multicenter Study

**DOI:** 10.1155/jonm/3947093

**Published:** 2026-02-01

**Authors:** Yannai DeJonghe, Herlinde Wynendaele, Els Clays, Jeroen Trybou

**Affiliations:** ^1^ Department of Public Health and Primary Care, Ghent University, Ghent, Belgium, ugent.be

**Keywords:** burnout, cross-sectional studies, nurses, nursing assistants, nursing homes, professional, quality of healthcare, working conditions

## Abstract

**Background:**

Growing demand for resident care has made it increasingly challenging for care workers (registered nurses and nursing assistants) to provide person‐centered and high‐quality care in nursing homes. Demanding working conditions, high workloads, and job‐related stress increase burnout risk among care workers. Positive work environments have been associated with reduced risk of emotional exhaustion and improved quality of care.

**Objectives:**

This study assessed the nursing home work environment, risk of burnout in care workers, and explored their association. Additionally, the study examined whether professional role moderates this association.

**Methods:**

A cross‐sectional, multicenter study included survey data from 819 care workers across 54 Belgian nursing homes. Linear mixed models examined associations between the work environment and burnout risk in care workers, with additional analyses exploring the moderating role of profession.

**Results:**

A substantial proportion of care workers reported positive collegial relationships (86.3%) and foundations for quality care (86.8%), while 65.5% reported insufficient staffing and resources. Additionally, 23.4% were at high risk of emotional exhaustion, 19.9% of depersonalization, and 32.2% of reduced personal accomplishment. All work environment measures were negatively associated with emotional exhaustion and depersonalization and positively associated with reduced personal accomplishment. Significant findings were obtained regarding the moderating role of profession.

**Conclusion:**

This study underscores the importance of improving the nursing home work environment. Implementing targeted interventions to enhance these elements could reduce burnout risk among care workers. While achieving adequate staffing levels is essential, the impact on care worker well‐being may be limited without additional improvements in the work environment. Granting care workers decision latitude and involving them in nursing home affairs could foster a more supportive and participative nursing management.

**Plain Language Summary**
• What is already known
◦ Globally, the need for long‐term care workers is rapidly increasing due to the growing proportion of senior citizens in each population, the advancing complexity of care, and their associated workload, challenging access to and the quality of long‐term care.
◦ Addressing this rising demand includes a more in‐depth understanding of the long‐term care workers’ working conditions and their well‐being, in order to formulate policies aimed at improving these aspects and reducing turnover.
◦ Prior research on the assessment of the nursing work environment, the risk of burnout, and their association has predominantly been conducted in hospital settings, often measuring only one dimension of burnout (emotional exhaustion).
• What this study adds
◦ Within our sample, 23.4% of nursing home care workers are at high risk of emotional exhaustion, 19.9% of depersonalization, and 32.2% of having a reduced sense of personal accomplishment.
◦ According to our findings, all measured nursing home work environment factors are negatively associated with the risk of emotional exhaustion and depersonalization and positively associated with a reduced sense of personal accomplishment.

## 1. Introduction

Long‐term care is increasingly recognized as a labor‐intensive service due to its significant physical and mental demands [1]. Currently, expanded opportunities within ambulatory care services and support of family and caregivers at home have led senior citizens to defer admission to a nursing home (NH). As a result of this delayed admission, trends show a reduced average length of stay in NHs and an elevated complexity in resident care needs [2]. Various elements, including higher prevalence of multimorbidity and greater limitations in physical and cognitive abilities, can be identified as contributing factors to the increasing complexity of care and associated workload [3].

With rising demands for resident care, providing person‐centered and high‐quality care has become an increasingly intricate and demanding task for registered nurses (RNs) and nursing assistants (NAs) (care workers) in NHs [4]. As a result of these precarious working conditions, high workloads, and job stresses, care workers in various healthcare settings have been found to be particularly at risk of burnout [5–7]. Burnout is a psychological work‐related stress syndrome caused by chronic exposure to job stress and is often characterized by emotional exhaustion (EE), depersonalization (DP), and reduced personal accomplishment (PA) [5, 6]. In addition, the effect of profession on the risk of burnout in care workers has been studied in a hospital setting, and findings have confirmed that not all professional categories are affected in the same manner [8]. Occupational burnout has proven to negatively affect personal distress, absenteeism, job turnover, and quality of care [6]. However, existing evidence regarding the multidimensional aspects of burnout in NH settings is limited, as prior research has predominantly focused on EE, often to the exclusion of DP and reduced PA.

Prior research has shown that positive work environments are associated with a reduced risk of EE in care workers and the delivery of high‐quality care [9–15]. In addition, according to Lake [16], this environment can be characterized by the presence of nursing foundations for quality care, capable and supportive managers, adequate staffing and resources, participation in organizational affairs, and positive collegial relationships.

The aging population is continuing to outpace the level of long‐term care workers [1]. In order to meet this increasing demand for care workers, policies regarding retention must be improved [17]. The work environment is one of the most prominent areas for interventions to improve care worker retention in NHs [14]. Moreover, it plays a pivotal role in their well‐being, implicating the need to thoroughly explore and provide in‐depth insights concerning the various aspects of the work environment, as well as its association to all three dimensions of burnout—not just EE. In addition, previous research has mainly been conducted in a hospital setting, and the limited evidence obtained from studies conducted in NHs is often inconclusive [14]. Despite the growing recognition of work environment factors in influencing the risk of burnout, the need for more comprehensive research within the NH setting remains. This study aims to fill the existing research gap by assessing the NH work environment and the risk of burnout in NH care workers and exploring their association by addressing the question: How do different work environment factors in NHs relate to various dimensions of burnout among care workers? We hypothesize that a more positive work environment will be associated with a lower risk of EE, DP, and reduced PA.

Secondarily, this study aims to provide insight into the role of profession in the association between work environment and risk of burnout in an NH setting by asking the question: Does the professional role moderate the association between work environment factors and burnout risk dimensions in NHs? We hypothesize that the professional role will moderate the association between NH work environment factors and the dimensions of burnout, and therefore results will differ based on the professional category of the care workers.

## 2. Materials and Methods

### 2.1. Study Setting, Design, and Sample

This is a cross‐sectional, multicenter study in Belgian NHs, reported according to the STrengthening the Reporting of OBservational studies in Epidemiology (STROBE) guidelines for cross‐sectional studies (Appendix A) [18]. NHs were allocated into subgroups based on inclusion criteria established for each of the following main characteristics: size, that is, small (less than 80 residents), medium (between 80 and 120 residents), and large (more than 120 residents); type of ownership, that is, public, not‐for‐profit, and for‐profit; and location, that is, the regions of Flanders, Wallonia, and Brussels. Based on these three main characteristics, a stratified random sample of 250 NHs was composed and invited to participate in the study. We opted for stratified random sampling on the NH‐level to help reduce sampling bias. Due to a notably low response rate, an additional stratified random sample of 112 NHs was added to the contact list. Overall, 362 Belgian NHs were contacted by our research team. A formal sample size calculation was not conducted, as the study was exploratory in nature and based on voluntary participation from NHs and their care workers.

Recruitment of care workers was coordinated in collaboration with a designated contact person within each participating NH. Following initial email invitations to sampled facilities, those expressing interest were invited to a digital information session, during which the research team outlined the study objectives, data collection procedures, and ethical safeguards, including informed consent, voluntary participation, and data protection measures. Upon agreement to participate, a staff member within the NH was appointed as the primary liaison. This contact person was responsible for disseminating the study information and participation invitation to eligible care workers, under the guidance of the research team. On the care worker‐level, the sample was obtained through convenience sampling.

Within participating NHs, all care workers providing direct resident care were contacted and invited to participate in the study as long as they were eligible. Inclusion criteria included RNs and NAs providing direct patient care for at least 1 month. Exclusion criteria encompassed new care workers to the organization (< 1 month), temporary employees, students, and those on extended leave (> 1 month). A survey instrument was developed and used to obtain data for this study, which can be found in Appendix B.

Our research utilized a multilevel study design to examine the predictors of burnout among NH care workers nested within different NHs. Each care worker (level 1) was considered within the context of their respective NH (level 2), recognizing the potential for correlated data within clusters. In total, we included complete survey data of 819 care workers from 54 NHs, of which 267 were RNs (90 holding a bachelor’s degree, 177 holding a diploma) and 552 were NAs. On the NH‐level, this corresponds with a response rate of 14.9% (54 out of 362 NHs contacted). In addition, 48 out of 54 participating NHs have reported the number of eligible care workers within their facility, which amounted to 1904. Within these 48 NHs, 784 care workers participated in our study, corresponding to a response rate of 41.2% on the care worker‐level.

### 2.2. Data Collection

Participants received a survey package a priori and voluntarily consented to participate in this study. The survey package included an information letter, details about the study and its purposes, data protection, and the right to not participate. The appointed contact person distributed the questionnaire, including an enclosed informed consent, to the eligible care workers.

Survey data were collected over a 4‐week period per NH, and reminders to complete the survey were sent on Weeks 2 and 3 in order to increase the response rate. Overall, data were collected online using Qualtrics between May 3^rd^ and December 24^th^ of 2021. Data were processed anonymously, and analyses were executed only at the NH‐level; thus, participants could not be identified. This study was approved by the Medical Ethics Committee of Ghent University (Hospital) (No. BC‐09667).

### 2.3. Measures

**FIGURE 1:**
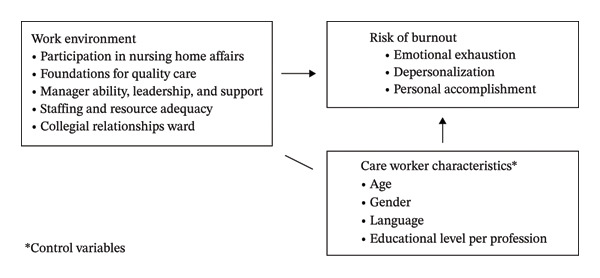
Overview measures.

An overview of all measures included in this study is provided in Figure 1, and elaborated upon in the following sections.

#### 2.3.1. Main Outcome: Risk of Burnout

The risk of burnout was measured using the Maslach Burnout Inventory (MBI), an instrument that has demonstrated high reliability and validity in past studies [6]. The instrument contains 22 items, which are presented in the form of statements regarding personal feelings or attitudes and can be allocated to one of three subscales. Certain items were slightly adjusted in wording in order to be applicable in an NH setting. A Cronbach’s alpha coefficient was computed for each subscale in order to determine its internal consistency and reliability: EE (*α* = 0.90), DP (*α* = 0.64), and reduced PA (*α* = 0.78) [19].

Participants were given a frequency scale ranging from 0 to 6, where 0 was defined as *never* and 6 as *every day*, and were asked to indicate how often they experienced the described feeling or attitude. As such, a score could be calculated per subscale. Subsequently, these scores could be categorized into one of three levels (low, moderate, or high), indicating the risk of burnout. Cutoff points for low, moderate, and high risk, were respectively, 0–18, 19–26, and > 26 for EE (score from 0 to 54); 0–5, 6–9, and > 9 for DP (score from 0 to 30); and > 39, 34–39, and 0–33 for reduced PA (score from 0 to 48) [6].

#### 2.3.2. Main Predictor: Work Environment

The work environment of the NH care workers was measured by the Practice Environment Scale of the Nursing Work Index (PES‐NWI), consisting of five subscales [16]. This instrument comprises 32 items, presented as statements and to be evaluated on a four‐point Likert scale (*strongly agree*, *agree*, *disagree*, and *strongly disagree*). Participants were asked to indicate to which degree each factor was present in their current work environment. In order to interpret the scores, a mean was calculated per subscale. In this four‐point response set, 2.5 was chosen as the neutral midpoint, meaning that all values above 2.5 indicated agreement with the given statement [16].

Given that this scale is typically used in hospital settings, we had to make some small adaptations to this validated instrument. These adaptations involved refining the wording of certain items to ensure their relevance in an NH setting. In order to assess the internal consistency of the multi‐item scales, a Cronbach’s alpha coefficient was computed for each subscale.

Firstly, the results from our own analysis will be shown, followed by those from the original survey instrument, which we added for comparison: participation in NH affairs (our *α* = 0.83; original *α* = 0.83), foundations for quality care (our *α* = 0.83; original *α* = 0.80), manager ability, leadership, and support (our *α* = 0.86; original *α* = 0.84), and staffing and resource adequacy (our *α* = 0.84; original *α* = 0.80) [16, 19].

Furthermore, we excluded the fifth subscale of the PES‐NWI, which measures the nurse–physician relations within the work environment. Considering the difference in both nature and intensity of work relations in care settings, we opted to include another scale to assess collegial relations within the work environment. The Safety Attitude Questionnaire (SAQ) Short Form includes items measuring the perceived quality of collaboration participants have experienced in the workplace with various colleagues, using a four‐point Likert scale (*strongly agree*, *agree*, *disagree*, and *strongly disagree*) [20]. In the SAQ Short Form, the quality of collaboration with three specified professions is assessed, namely nurses, physicians, and pharmacists. In order to adequately evaluate all forms of collaboration in an NH setting, we have expanded this list to 18 professions (e.g., colleagues in the ward, head of the ward, allied health professionals). For this analysis, we only included the item assessing collegial relationships in the ward.

#### 2.3.3. Covariates: Care Worker Characteristics

For analytic purposes, we identified and controlled for several covariates, which can be described as care worker characteristics. In Belgium, two profound communities can be identified based on language, namely a Dutch‐ and French‐speaking community. We found that “language” correlated more strongly to the main outcome variable compared to “region” and have thus included “language” as a covariate in our multilevel regression analyses. In addition, care worker characteristics “gender” and “age” (18–30; 31–40; 41–50; 51–60; older than 60) were also identified as covariates. Furthermore, we defined “profession” as a final covariate in these analyses, for which we combined two care worker characteristics: “professional category” (RN; CA) and “educational level” (diploma; bachelor’s degree). In Belgium, NAs are most likely holders of a diploma, whereas RNs can be holders of a diploma as well as a bachelor’s degree. For this reason, we decided to make a distinction between RNs with a bachelor’s degree (bachelor RNs) and RNs with a diploma (basic RNs).

### 2.4. Statistical Analysis

At first, descriptive statistics were generated to examine the NH care worker and NH characteristics. Next, assumptions regarding normality, homoscedasticity, and multicollinearity were assessed before model fitting. Pearson’s correlation coefficients between the predictor variables were computed, for which we established a multicollinearity threshold of *r* = 0.8. We concluded that most measures for work environment correlated too highly with one another, which led us to build several multilevel linear mixed models to investigate their associations with risk of burnout separately. Subsequently, care worker characteristics (age, gender, language, and profession; measured at the individual care worker level) were added to the linear mixed models as control variables.

As our primary interest was to explore individual‐level predictors, we did not include higher‐level (NH) variables. However, each care worker (level 1) was considered within the context of their respective NH (level 2), accounting for the nested character of our obtained survey data and the potential for correlated data within these clusters. In addition, intraclass correlation coefficients (ICCs) are reported to quantify the proportion of variance attributable to differences between NHs versus within NHs across care workers.

Five separate 2‐level linear mixed models were specified to assess the association between each work environment subscale (participation in NH affairs; foundations for quality care; manager ability, leadership, and support; staffing and resource adequacy; teamwork and safety climate) and the risk of burnout, while also controlling for individual care worker characteristics. These models included fixed effects for the work environment subscales and the individual‐level covariates, as well as random intercepts to account for the clustering of care workers within NHs. The multilevel models were estimated using restricted maximum likelihood (REML), which is appropriate for our nested data structure. The covariance structure for random effects was assumed to be variance components, treating each NH as a random sample from a larger population.

This process was repeated once for every subscale of the main outcome variable, given that all three subscales assess different aspects of the experienced risk of burnout [6]. Consequently, a multiple‐comparison correction was performed to account for the multiple testing problem. The Bonferroni method was applied to the number of statistical analyses performed simultaneously (*n* = 15), which resulted in an adjusted alpha level of *p* < 0.003 [21].

In addition, moderation analyses of all covariates were performed by adding the moderator and interaction term (moderator∗predictor) to the existing linear mixed model exploring the association between the predictor and outcome variable [22]. The addition of the variable “profession” as a moderator to the beforementioned linear mixed model resulted in multiple statistically significant interaction effects. Therefore, an additional stratified analysis based on “profession” was performed, exploring the differences in results between bachelor RNs, basic RNs, and NAs. All data analyses were conducted using SPSS Version 29, using the GENLINMIXED procedure. The proportion of missing values across key variables was relatively low, ranging from 0% to 8.5%, with an average of 4.3%. More specifically, 8.3% of data were missing for participation in NH affairs and foundations for quality care. Missing data for manager ability, leadership, and support and staffing and resource adequacy were estimated at 8.5%, and 2% for collegial relationships in the ward. The analysis showed 0%–0.1% of missing data for EE, DP, and reduced PA. Furthermore, we found that missing data for all care worker characteristics ranged between 0% and 2.9%, with an average of 0.95%. Due to relatively lower levels of missingness, missing data were excluded from analysis through listwise deletion across all models.

## 3. Results

### 3.1. Descriptive Data

Overall, 54 NHs and 819 care workers were included in this study. Table 1 summarizes NH‐ and individual care worker‐level characteristics, as well as individual‐level results for work environment measures and risk of burnout outcomes. It shows that the majority of NHs are situated in Flanders, comprising 59.3% of the total sample. Wallonia follows, representing 35.2%, while Brussels accounts for a smaller percentage at 5.6%. In addition, small‐sized facilities constitute the largest proportion at 38.9%. Publicly owned facilities comprise the largest share at 42.6%. Private for‐profit and not‐for‐profit entities hold a slightly smaller portion of this sample at 31.5% and 25.9%, respectively. The majority of care workers were female (92%) and spoke Dutch (68.9%). On average, our participants were 41.8 years old. In our sample, 32.6% were RNs, of which 11% hold a bachelor’s degree.

**TABLE 1:** NH‐ and individual care worker‐level characteristics, and individual‐level results for work environment and risk of burnout outcomes.

	Sample	Burnout‐EE (*n* = 819)	Burnout‐DP (*n* = 818)	Burnout‐PA (*n* = 818)
	*n* (%)	*n* (%) high risk	*n* (%) high risk	*n* (%) high risk
Nursing home‐level (*n* = 54)				
Region				
Flanders (*n* = 561, 561, 560)	32 (59.3)	124 (22.1)	102 (18.2)	155 (27.7)
Wallonia (*n* = 218, 217, 218)	19 (35.2)	63 (28.9)	57 (26.3)	89 (40.8)
Brussels (*n* = 40)	3 (5.6)	5 (12.5)	4 (10)	19 (47.5)
Size				
Small (*n* = 169)	21 (38.9)	38 (22.5)	36 (21.3)	63 (37.3)
Medium (*n* = 293, 292, 293)	16 (29.6)	70 (23.9)	58 (19.9)	90 (30.7)
Large (*n* = 357, 357, 356)	17 (31.5)	84 (23.5)	69 (19.3)	110 (30.9)
Type of ownership				
Public (*n* = 432; 431; 431)	23 (42.6)	105 (24.3)	93 (21.6)	137 (31.8)
Private not‐for‐profit (*n* = 258)	14 (25.9)	49 (19)	39 (15.1)	82 (31.8)
Private for‐profit (*n* = 129)	17 (31.5)	38 (29.5)	31 (24.0)	44 (34.1)
Individual‐level (*n* = 819)				
Gender (*n* = 812)				
Female (*n* = 747, 747, 746)	747 (92)	176 (23.6)	149 (19.9)	238 (31.9)
Male (*n* = 61, 60, 61)	61 (7.5)	15 (24.6)	13 (21.7)	16 (26.2)
Other (*n* = 4)	4 (0.5)	0 (0)	0 (0)	3 (75)
Age groups in years (*n* = 795)				
18–30 (*n* = 188, 187, 188)	188 (23.6)	60 (31.9)	54 (28.9)	57 (30.3)
31–40 (*n* = 165)	165 (20.8)	31 (18.8)	32 (19.4)	54 (32.7)
41–50 (*n* = 218, 218, 217)	218 (27.4)	48 (22)	45 (20.6)	78 (35.9)
51–65 (*n* = 224)	224 (28.2)	50 (22.3)	28 (12.5)	65 (29)
Age in years, mean (SD)	41.8 (11.8)			
Language				
Dutch (*n* = 564, 564, 563)	564 (68.9)	124 (22)	102 (18.1)	157 (27.9)
French (*n* = 255, 254, 255)	255 (31.1)	68 (26.7)	61 (24)	106 (41.6)
Profession				
Bachelor RN (*n* = 90)	90 (11)	19 (21.1)	16 (17.8)	29 (32.2)
Basic RN (*n* = 177, 176, 177)	177 (21.6)	42 (23.7)	34 (19.3)	58 (32.8)
Nursing assistants (*n* = 552, 552, 551)	552 (67.4)	131 (23.7)	113 (20.5)	176 (31.9)
	***n* (%)**	**Mean (SD)**		

Work environment				
Participation in nursing home affairs (*n* = 751)	441 (58.7)^†^	2.59 (0.58)		
Foundations for quality care (*n* = 751)	652 (86.8)^†^	2.96 (0.46)		
Manager ability, leadership, and support (*n* = 749)	522 (69.7)^†^	2.84 (0.68)		
Staffing and resource adequacy (*n* = 749)	258 (34.4)^†^	2.28 (0.66)		
Collegial relationships ward (*n* = 803)	693 (86.3)^‡^	3.15 (0.68)		
Risk of burnout				
Emotional exhaustion (*n* = 819)	192 (23.4)^§^	18.13 (12.19)		
Depersonalization (*n* = 818)	163 (19.9)^§^	5.28 (5.48)		
Reduced personal accomplishment (*n* = 818)	263 (32.2)^§^	36.18 (8.39)		

*Note:* Scale ranges: work environment 1–4; EE 0–53; DP 0–27; PA 0–48.

Abbreviations: DP, depersonalization; EE, emotional exhaustion; PA, personal accomplishment.

^†^Number of participants (and percentage) in (total) agreement with correlating items of this PES‐NWI subscale.

^‡^Number of participants (and percentage) who assessed this item as good or excellent.

^§^Number of participants (and percentage) with a high risk of burnout within this MBI subscale.

A large proportion of the participants indicated having good or excellent collegial relationships in their ward (86.3%) and found themselves agreeing with items measuring the presence of nursing foundations for quality care (86.8%) in their work environment. On the contrary, only one‐third of the participants (34.4%) have indicated having adequate staff and resources to provide quality care. Furthermore, a substantial proportion of care workers assessed their manager’s abilities, leadership, and support (69.7%) in a positive manner and indicated having a participatory role within their NH’s affairs (58.7%). When assessing the risk of burnout within our participants, we found 23.4% to have a high risk of experiencing EE, 19.9% encountering feelings of DP, and 32.2% having a reduced sense of PA.

Through descriptive observation, we found that small NHs have slightly higher percentages of care workers at high risk of experiencing a reduced sense of PA compared to medium and large NHs in our sample (37.3%; 30.7%; 30.9%). In addition to these observations, private for‐profit NHs in our sample appear to have a larger proportion of care workers at high risk of burnout compared to private not‐for‐profit NHs in our sample, particularly regarding the experience of EE (29.5% vs. 19.0%; DP 24.0% vs. 15.1%; RPA 34.1% vs. 31.8%). We also found more care workers at high risk of EE (31.9%) and DP (28.9%) in the age category of 18–30, as opposed to the other age groups. Furthermore, our findings show a higher percentage of French‐speaking care workers at high risk of burnout compared to those speaking Dutch, especially for reduced PA (EE 26.7% vs. 22%; DP 24% vs. 18.1%; RPA 41.6% vs. 27.9%).

### 3.2. Associations Between Work Environment and Risk of Burnout

Assumptions of normality and homoscedasticity were assessed via standardized conditional residual histograms, normal Q–Q plots, and residuals‐versus‐fitted plots. Although mild tail deviations were observed, simulation studies demonstrate that LMMs remain robust to such violations under moderate to large sample sizes [23]. Table 2 summarizes our findings with respect to the association between work environment measures and risk of burnout outcomes. Data analysis was performed through the execution of several linear mixed models at the individual care worker‐level, each testing an association between one work environment measure and one of three risk of burnout outcomes. While controlling for several care worker characteristics (age, gender, language, and profession), we found all work environment measures to be negatively associated (*p < *0.001) with the risk of EE and DP. For example, for each one‐unit increase in the mean score of the subscale “participation in NH affairs,” the EE score decreases by 8.89 points on average (*β = *−8.89; 95% *CI*: −10.33 to −7.45), while holding other variables constant. Furthermore, all work environment predictors were positively associated (*p < *0.001) with the risk of reduced PA. For example, for each one‐unit increase in the mean score of the subscale “participation in NH affairs,” the reduced PA score increases by 2.28 points on average (*β = *2.28; 95% *CI*: 1.23–3.32), while holding other variables constant. The reduced PA subscale is a scale where higher scores indicate lower burnout risk. Therefore, this positive association suggests that higher participation in NH affairs is linked with a lower risk of reduced PA, reflecting a decrease in burnout risk.

**TABLE 2:** Associations between work environment measures and risk of burnout outcomes, multilevel analyses.

Work environment measures	Burnout‐EE (*n* = 819)				Burnout‐DP (*n* = 818)				Burnout‐PA (*n* = 818)			
	*β*		95% CI		*β*		95% CI		*β*		95% CI	
Participation in nursing home affairs (*n* = 726, 725, 725)	−8.89^∗^		(−10.33, −7.45)		−3.53^∗^		(−4.19, −2.88)		2.28^∗^		(1.23, 3.32)	
Foundations for quality care (*n* = 726, 725, 725)	−11.08^∗^		(−12.86, −9.29)		−4.50^∗^		(−5.31, −3.69)		3.07^∗^		(1.77, 4.36)	
Manager ability, leadership, and support (*n* = 724, 723, 723)	−7.62^∗^		(−8.88, −6.37)		−2.74^∗^		(−3.31, −2.16)		2.19^∗^		(1.30, 3.09)	
Staffing and resource adequacy (*n* = 724, 723, 723)	−9.20^∗^		(−10.46, −7.94)		−2.97^∗^		(−3.57, −2.38)		1.71^∗^		(0.76, 2.66)	
Collegial relationships ward (*n* = 779, 778, 778)	−4.32^∗^		(−5.52, −3.12)		−1.23^∗^		(−1.79, −0.68)		1.77^∗^		(0.94, 2.61)	

**Model fit statistics**	**R** ^2^ **cond.**	**ICC**	**AIC**	**BIC**	**R** ^2^ **cond.**	**ICC**	**AIC**	**BIC**	**R** ^2^ **cond.**	**ICC**	**AIC**	**BIC**

Participation in nursing home affairs	0.25	0.06	5507.8	5516.9	0.21	0.04	4382.2	4391.3	0.10	0.04	5054.0	5063.1
Foundations for quality care	0.25	0.06	5506.7	5515.8	0.21	0.03	4376.6	4385.7	0.10	0.04	5050.4	5059.5
Manager ability, leadership, and support	0.25	0.06	5496.4	5505.5	0.18	0.04	4392.5	4401.7	0.10	0.04	5028.7	5037.8
Staffing and resource adequacy	0.29	0.04	5453.0	5462.1	0.19	0.04	4384.1	4393.2	0.09	0.04	5047.3	5056.4
Collegial relationships ward	0.17	0.11	5992.7	6002.0	0.12	0.07	4792.6	4801.8	0.10	0.05	5426.1	5435.4

*Note:* All models include a random intercept for nursing home and were adjusted for covariates at the individual level (age, gender, language, and profession).

Abbreviations: DP, depersonalization; EE, emotional exhaustion; PA, personal accomplishment.

^∗^ *p* < 0.001.

The presence of foundations for quality care (*β* = −11.08; 95% *CI* −12.86, −9.29; *ICC* 0.06), obtaining adequate staffing levels and resources (*β* = −9.20; 95% *CI* −10.46, −7.94; *ICC* 0.04), being able to participate in NH affairs (*β* = −8.89; 95% *CI* −10.33, −7.45; *ICC* 0.06), and manager ability, leadership, and support (*β* = −7.62; 95% *CI* −8.88, −6.37; *ICC* 0.06) have the strongest effects in regard to decreasing the risk of EE. Notably, the ICC for collegial relationships in the ward (*β* = −4.32; 95% *CI* −5.52, −3.12; *ICC* 0.11) indicates that a higher percentage of variance in risk of EE can be attributed to differences between NHs, as opposed to the other work environment measures.

Furthermore, the moderation analysis revealed multiple statistically significant results for profession as a moderator in regard to the association between work environment and risk of burnout, which are summarized in Table 3. As a result, the effect of this moderator on associations between work environment measures and risk of burnout was further explored. These additional linear mixed models examined the differences in results based on profession, which are shown in Table 4. The analysis was adjusted for several care worker characteristics (age, gender, and language), and results were stratified based on “profession.”

**TABLE 3:** Moderation analysis for profession in regard to associations between work environment subscales and risk of burnout outcomes.

Work environment measures	Burnout‐EE (*n* = 819)		Burnout‐DP (*n* = 818)		Burnout‐PA (*n* = 818)	
	*F*	*α*	*F*	*α*	*F*	*α*
Main and interaction effects						
Participation in nursing home affairs (*n* = 751, 750, 750)						
Subscale	95.05	< 0.001	89.16	< 0.001	13.55	< 0.001
Profession	0.63	0.534	2.88	0.057	0.49	0.616
Subscale × profession	0.89	0.411	3.64	0.027	0.48	0.617
Foundations for quality care (*n* = 751, 750, 750)						
Subscale	94.07	< 0.001	72.88	< 0.001	11.41	< 0.001
Profession	0.37	0.693	0.63	0.532	1.15	0.318
Subscale × profession	0.54	0.584	0.89	0.410	1.01	0.366
Manager ability, leadership, and support (*n* = 749, 748, 748)						
Subscale	88.21	< 0.001	60.42	< 0.001	21.95	< 0.001
Profession	0.03	0.973	0.82	0.441	1.34	0.261
Subscale × profession	0.03	0.971	1.14	0.321	1.44	0.238
Staffing and resource adequacy (*n* = 749, 748, 748)						
Subscale	150.10	< 0.001	78.56	< 0.001	12.29	< 0.001
Profession	3.09	0.046	2.76	0.064	0.73	0.483
Subscale × profession	3.71	0.025	3.34	0.036	0.77	0.463
Collegial relationships ward (*n* = 803, 802, 802)						
Subscale	33.46	< 0.001	14.65	< 0.001	13.89	< 0.001
Profession	0.72	0.488	0.90	0.405	1.12	0.328
Subscale × profession	0.77	0.465	1.15	0.317	1.00	0.369

Abbreviations: DP, depersonalization; EE, emotional exhaustion; PA, personal accomplishment.

**TABLE 4:** Associations between work environment measures and risk of burnout outcomes, stratified on profession.

	Burnout‐EE (*n* = 819)		Burnout‐DP (*n* = 818)		Burnout‐PA (*n* = 818)	
	*β*	95% CI	*β*	95% CI	*β*	95% CI
Work environment measures						
Participation in nursing home affairs						
Bachelor RNs (*n* = 74)	−7.67^∗^	(−12.23, −3.10)	−5.04^∗^	(−7.06, −3.03)	3.01^+^	(0.13, 5.89)
Basic RNs (*n* = 160, 159, 160)	−9.13^∗^	(−12.23, −6.04)	−4.34^∗^	(−5.73, −2.96)	3.65^∗∗^	(1.33, 5.98)
NAs (*n* = 492, 492, 491)	−8.89^∗^	(−10.62, −7.16)	−3.11^∗^	(−3.91, −2.31)	2.03^∗∗^	(0.76, 3.30)
Foundations for quality care						
Bachelor RNs (*n* = 74)	−12.48^∗^	(−18.41, −6.56)	−5.43^∗^	(−8.29, −2.56)	2.04	(−1.70, 5.79)
Basic RNs (*n* = 160, 159, 160)	−10.31^∗^	(−14.42, −6.20)	−5.22^∗^	(−7.06, −3.37)	5.09^∗^	(2.09, 8.08)
NAs (*n* = 492, 492, 491)	−11.17^∗^	(−13.27, −9.08)	−4.29^∗^	(−5.24, −3.34)	2.93^∗^	(1.39, 4.47)
Manager ability, leadership, and support						
Bachelor RNs (*n* = 74)	−6.34^∗^	(−9.95, −2.73)	−2.71^∗∗^	(−4.45, −0.96)	3.18^∗∗∗^	(0.95, 5.42)
Basic RNs (*n* = 160, 159, 160)	−5.81^∗^	(−8.74, −2.88)	−3.29^∗^	(−4.56, −2.01)	4.03^∗^	(1.99, 6.07)
NAs (*n* = 490, 490, 489)	−8.29^∗^	(−9.74, −6.83)	−2.64^∗^	(−3.32, −1.95)	1.75^∗∗^	(0.67, 2.84)
Staffing and resource adequacy						
Bachelor RNs (*n* = 74)	−12.91^∗^	(−16.86, −8.96)	−5.04^∗^	(−7.05, −3.03)	2.16	(−0.46, 4.77)
Basic RNs (*n* = 160, 159, 160)	−9.90^∗^	(−12.95, −6.84)	−3.79^∗^	(−5.18, −2.39)	3.53^∗∗^	(1.22, 5.83)
NAs (*n* = 490, 490, 489)	−8.66^∗^	(−10.12, −7.20)	−2.57^∗^	(−3.27, −1.88)	1.52^∗∗∗^	(0.41, 2.63)
Collegial relationships ward						
Bachelor RNs (*n* = 90)	−4.27^+^	(−7.82, −0.71)	−1.31	(−3.10, 0.49)	2.32	(−0.33, 4.96)
Basic RNs (*n* = 169, 168, 169)	−5.02^∗^	(−7.86, −2.18)	−2.03^∗∗^	(−3.36, −0.71)	3.51^∗^	(1.46, 5.57)
NAs (*n* = 520, 520, 519)	−4.13^∗^	(−5.56, −2.69)	−1.06^∗^	(−1.71, −0.41)	1.43^∗∗∗^	(0.45, 2.41)

*Note:* All models include a random intercept for nursing home and were adjusted for covariates at the individual level (age, gender, and language), analysis was stratified based on profession.

Abbreviations: DP, depersonalization; EE, emotional exhaustion; NAs, nursing assistants; PA, personal accomplishment; RNs, registered nurses.

^+^ *p* ≤ 0.05.

^∗^ *p* ≤ 0.001.

^∗∗^ *p* ≤ 0.003.

^∗∗∗^ *p* ≤ 0.01.

Statistically significant interaction effects of profession were found in the negative association between staffing and resource adequacy and the risk of both EE and DP. More particularly, obtaining adequate staffing levels and resources has a stronger decreasing effect on the risk of EE for bachelor RNs (*β* = −12.91; 95% *CI* −16.86, −8.96), compared to basic RNs (*β* = −9.90; 95% *CI* −12.95, −6.84) and NAs (*β* = −8.66; 95% *CI* −10.12, −7.20). We established similar findings for the risk of DP when comparing results for bachelor RNs (*β* = −5.04; 95% *CI* −7.05, −3.03) to basic RNs (*β* = −3.79; 95% *CI* −5.18, −2.39) and NAs (*β* = −2.57; 95% *CI* −3.27, −1.88). In addition, a statistically significant interaction effect of profession was observed in the negative association between participation in NH affairs and the risk of DP. Regarding this association, we found a slightly stronger decreasing effect in the risk of DP when bachelor RNs (*β* = −5.04; 95% *CI* −7.06, −3.03) and basic RNs (*β* = −4.34; 95% *CI* −5.73, −2.96) are able to participate in NH affairs, compared to NAs (*β* = −3.11; 95% *CI* −3.91, −2.31).

## 4. Discussion

The primary aim of this study was to assess the NH work environment and the risk of burnout in care workers and to explore their association. While previous studies predominantly focused on hospital environments, this research addresses the work environment and multiple burnout dimensions within NH care settings. According to Lake’s findings [16], a positive work environment can be defined by the presence of nursing foundations for quality care, capable and supportive managers, adequate staffing and resources, participation in organizational affairs, and positive collegial relationships. In this study, the majority of participants reported positive collegial relationships (86.3%) and affirmed the presence of nursing foundations for quality care (86.8%) in their work environment. These findings are consistent with prior research in Belgian hospitals and international research [24–26]. However, the majority of RNs and NAs also indicate having insufficient staff and resources (65.6%) to deliver quality care. Previous research has provided insights on low staffing levels and their impact on the delivery of quality care and the well‐being of care workers [27–30]. Aligned with these insights, our assessment shows a substantial proportion of NH care workers at high risk of burnout (23.4% EE, 19.9% DP, and 32.2% reduced PA).

Controlled for several care worker characteristics (age, gender, language, and profession), this study found (1) participation in NH affairs; (2) foundations for quality care; (3) manager ability, leadership, and support; (4) staffing and resources adequacy; and (5) collegial relationships in the NH ward to be negatively associated (*p* < 0.001) with the risk of EE and DP and positively associated (*p* < 0.001) with reduced PA. Our findings align with those of Chiminelli‐Tomás et al., who reviewed existing literature and found similar associations in hospital settings, though this study extends the evidence to NHs [31].

Previous research has provided evidence to support the hypothesis that NH care workers in good work environments are less likely to be at risk of EE [13, 14]. Our findings confirm the association between measures for a positive work environment and the risk of burnout in NH care workers, not only for EE but for DP and reduced PA as well.

The secondary aim was to provide insight into the role of profession in the association between work environment and risk of burnout in an NH setting. A moderation analysis, controlled for several care worker characteristics (age, gender, and language), revealed several statistically significant results regarding the role of the profession as a moderator in the association between work environment and the risk of burnout. More specifically, the presence of adequate staffing levels and resources was observed to have a stronger decreasing effect on the risk of both EE and DP in bachelor RNs compared to basic RNs and NAs. Overall, staffing levels of bachelor RNs employed in NH settings are considered to be relatively low [32]. This phenomenon can be explained by the fact that employment in NHs is often less appealing to bachelor RNs due to their association with poorer working conditions, high physical and mental risks, a lower career status, and reduced salaries [33, 34]. Furthermore, research by Chau et al. [35] shows that bachelor nursing education graduates have better critical‐thinking, reasoning, and organizational skills, which can lead to a broader understanding of practice guidelines concerning the organization of care, such as staffing and resource policies. This might suggest that the professional role and scope of practice of this group amplifies their sensitivity to organizational conditions, as they typically carry greater clinical responsibility and accountability for decision‐making, as well as more frequent involvement in care planning and coordination [14]. In fact, prior research focusing on RNs has stated that the risk of burnout is strongly related to factors such as mental work overload and role stress, which are exacerbated by insufficient staffing levels [36, 37].

These lower staffing levels of bachelor RNs, combined with their broader understanding of organization of care and heavier burden of responsibility, could explain the stronger effect of staffing and resource adequacy on their experienced risk of EE and DP.

Furthermore, we observed slightly stronger results for RNs regarding the association between participation in NH affairs and the risk of DP compared to NAs. Previous research has suggested that involvement of RNs in the decision‐making process can have a mediating role in the association between work environment and risk of burnout, which can be confirmed by our findings [38]. Moreover, Senol Çelik et al. found that both structural and psychological empowerment can significantly reduce burnout levels in nurses, specifically noting a moderate and negative association between structural empowerment and the risk of DP and job commitment [39]. Other research identified low decision latitude, low control over the job, low autonomy, low structural empowerment, and low participation in facility affairs as contributors to burnout in nurses working in hospital settings [40]. Additionally, self‐determination and autonomy, psychological empowerment, and job involvement in facility affairs and nursing policy development have also been repeatedly associated with job satisfaction and retention of RNs working in NHs [41, 42]. These results emphasize the importance of care worker autonomy and structural empowerment in their professional roles and their involvement in nursing policy and participation in NH affairs [39, 41, 42].

### 4.1. Limitations

Due to the cross‐sectional design, causality cannot be established between work environment factors and burnout outcomes. As a result, our study was limited to exploring associations rather than causal relationships.

While the conceptual model assumes that characteristics of the work environment influence care workers’ risk of burnout, the reverse may also be true: Care workers experiencing higher levels of burnout may perceive their work environment more negatively, potentially introducing reverse causation. This possibility should be acknowledged when interpreting the findings. Longitudinal or intervention studies are needed to assess the directionality and potential reciprocal relationships between work environment and burnout. As all variables were self‐reported at a single time point, there is a potential for common method bias. This reliance on a common measurement method may have inflated associations between variables. However, several factors mitigate this concern, including the use of validated instruments and assurances of anonymity to reduce social desirability bias. Future research would benefit from multimethod assessment approaches to minimize shared method variance [43].

Across all key variables, the proportion of missing values was relatively low, ranging from 0.0% to 8.5% (4.3% on average). Therefore, listwise deletion was applied to handle missing data, which was considered appropriate given the limited extent of missingness, and allowed for consistent case inclusion across models. Nonetheless, the potential impact on generalizability should be considered when interpreting the results. Although a fair amount of NH care workers (*n* = 819) participated in the study, the response rate at the NH level was rather low. During a 4‐month period, we sampled and contacted 362 NHs in Belgium. Despite our dedicated efforts to augment participation, the response rate remained low, with only 14.9% (*n* = 54) of the contacted NHs engaging in our study. This rather low response rate might possibly introduce nonresponse bias at the facility level. To explore this, we conducted three chi‐square tests comparing participating and nonparticipating NHs on the key organizational characteristics used in our stratified sampling approach: region, type of facility, and size. A Bonferroni correction was applied to adjust for multiple comparisons (adjusted significance threshold: *p* < 0.017).

The analyses revealed no statistically significant differences in region (*p* = 0.275) or size (*p* = 0.659), suggesting comparability across these characteristics. However, a significant difference was observed for facility type (*p* = 0.009), indicating that certain types of NHs may have been more likely to participate. This introduces a potential source of nonresponse bias, which may limit the generalizability of the findings. Therefore, results should be interpreted with caution. We identified two primary impediments to participation. Firstly, the COVID‐19 pandemic contributed to heightened staff absenteeism and increased workloads, leading NHs to deem study participation unfeasible at the time.

Secondly, NHs and their care workers conveyed their participation in an excessive number of studies, attributing this to the development of survey fatigue among their care workers [44]. The development of survey fatigue is hypothesized to stem from an increased reliance on survey‐based research, in this case caused by the COVID‐19 pandemic, and is characterized by diminished response rates. The individual‐level response rate of 41.2% within 48 NHs in our sample might be an indication of the presence of survey fatigue among the NH care workers, suggesting that they experienced a sense of being overwhelmed by the numerous survey requests, ultimately leading to a notable increase in nonresponses.

### 4.2. Implications and Future Research

This study underlines the importance of improving the work environment in NHs, focusing on factors such as (1) participation in NH affairs; (2) foundations for quality care; (3) manager ability, leadership, and support; (4) staffing and resource adequacy; and (5) collegial relationships in the NH ward. Implementing interventions to enhance these elements could contribute to reducing the risk of EE, DP, and reduced PA among NH care workers. According to our findings, the majority of NH care workers have assessed the staffing levels and resources to be inadequate.

The long‐term care sector encounters some challenges regarding recruitment and retention, such as low wages, high physical and mental risks, and limited access to training, education, and career prospects [34]. Obtaining sufficient resources and appropriate staffing ratios could reduce workload stress and enhance care quality [45]. Additionally, continuous education and career development opportunities contribute to a strong professional identity, which also mitigates the risk of burnout and turnover intentions [46]. Establishing more positive working conditions alongside promoting education, training, and career development opportunities, as well as enhancing professional role clarity, could contribute to making employment in NHs more attractive to care workers, which might improve both retention and recruitment [33, 34, 42]. For example, the role of RNs could be further differentiated in NH settings in order to improve retention. The basic RNs could be considered to play a key role in direct resident care, performing specific nursing tasks (e.g., administering medication), acting as case managers of residents, and being the first point of contact for them and other care workers. As a result, the focus of bachelor RNs could be redirected to indirect resident care tasks, such as taking on a coaching role, stimulating evidence‐based practices, or implementing nursing policies [42]. In addition, the COVID‐19 pandemic necessitated substantial reorganization of care within long‐term care facilities. In response to acute staffing shortages and increased care demands, several governments temporarily modified licensing and credentialing regulations, thereby enabling task‐shifting from RNs to CAs, or even to nontraditional NH staff. While these emergency measures were initially conceived as short‐term solutions, they underscore the importance of re‐evaluating workforce models. Translating selected temporary adjustments into sustainable policy innovations may help mitigate structural staffing shortages and enhance resilience in long‐term care systems moving forward [47].

However important, the impact of solely increasing staffing levels on the well‐being of care workers may be limited, unless other aspects of a positive work environment are also established [14]. For instance, care workers should be actively involved in decision‐making processes, extending from daily nursing tasks to broader NH strategies. This includes active participation in resident care plans and multidisciplinary meetings, as well as contribution to the development of NH policies and quality improvement initiatives [38, 48, 49]. Increasing the direct involvement of care workers in NH affairs does not only validate their professional role and expertise, it also allows for relevant, practice‐based input regarding areas of improvement in the work environment [50]. Providing care workers with decision latitude, promoting engagement, and increasing their involvement in NH affairs can foster a more participative and supportive nursing management style [38, 48, 49]. An important aspect of this approach is direct leadership and care worker empowerment, which can be characterized by some of the following good practices: two‐way communication, involvement in decision‐making, support, education and coaching, and practices that communicate that care workers are valued [49, 51, 52]. Ultimately, NH managers have an important role to fulfill in terms of providing the foundations, support, and adequate resources for a positive work environment. [52]. This highlights the need for support, education, and continuous professional development of NH managers. Facilitating management training regarding their professional roles and leadership strategies could aid NH managers in the development of targeted interventions contributing to a positive and supportive work environment, which in turn could improve the well‐being of care workers [40, 53].

Finally, longitudinal research is essential to establish causality and confirm directionality between the NH work environment and the risk of burnout in care workers, providing a deeper understanding of this relationship. Additionally, it is important to assess the effectiveness and long‐term impact of interventions aimed at improving the NH work environment, and determine whether these positive changes lead to reduced burnout and enhanced well‐being among its care workers.

## 5. Conclusions

In conclusion, this cross‐sectional study aimed to evaluate the NH work environment, assess the risk of burnout among NH care workers, and explore the intricate associations between these factors. The majority of participants indicated having positive collegial relationships and affirmed the presence of nursing foundations for quality care in their work environment. However, a substantial proportion of RNs and NAs reported insufficient staffing and resources to provide quality care. After adjusting for care worker characteristics, this study identified several work environment factors that have statistically significant associations with the risk of burnout. According to our findings, (1) participation in NH affairs; (2) foundations for quality care; (3) manager ability, leadership, and support; (4) staffing and resources adequacy; and (5) collegial relationships in the NH ward are negatively associated with the risk of EE and DP and positively associated with a reduced sense of PA. The results of this study suggest that all included factors contributing to a more positive work environment in NHs are associated with a lower risk of burnout in care workers within our sample. These findings underscore the need for targeted policies to improve the NH work environment, which might positively impact the risk of burnout in care workers. However, longitudinal research is crucial to explore causal pathways and the long‐term impact of these work environment interventions.

This study also contributes valuable insights in regard to the role of profession in the association between work environment and risk of burnout in an NH setting. Our findings show stronger outcomes for bachelor RNs in the association between staffing and resource adequacy and the risk of EE, as well as DP. In addition, we found a similar result for RNs in the association between participation in NH affairs and the risk of DP.

## Author Contributions

Yannai DeJonghe: conceptualization, formal analysis, investigation, methodology, and writing–original draft. Herlinde Wynendaele: investigation and writing–review and editing. Els Clays: conceptualization, methodology, supervision, and writing–review and editing. Jeroen Trybou: conceptualization, methodology, funding acquisition, supervision, and writing–review and editing.

## Funding

This research was supported by the Belgian Health Care Knowledge Centre (10.13039/501100019353), an independent research institution funded by the federal government.

## Disclosure

The authors would like to acknowledge that an earlier draft of this manuscript was accepted for presentation at the Annual Meeting of the Academy of Management in Chicago, Illinois (August 9–13, 2024). The conference abstract was published online on July 9, 2024, and in print on August 1, 2024, in the *Academy of Management Annual Meeting Proceedings*, which includes abstracts of all studies and symposia presented at the annual conference (https://doi.org/10.5465/AMPROC.2024.20249abstract). The funding source had no influence on the data collection, analysis, interpretation, or the decision to submit the results for publication.

## Conflicts of Interest

The authors declare no conflicts of interest.

## Supporting Information

Supporting Information for this article includes STrengthening the Reporting of OBservational studies in Epidemiology (STROBE) guidelines for cross‐sectional studies checklist, which outlines the reporting standards followed in this study, and an overview of the survey instruments used for data collection. Both documents are available to provide additional transparency and detail regarding the study’s methodology and adherence to reporting guidelines.

## Supporting information


**Supporting Information** Additional supporting information can be found online in the Supporting Information section.

## Data Availability

The anonymized data and code that support the findings of this study are available from the corresponding author upon reasonable request.
