# Supplementary material for: Nursing Home Work Environment and Burnout Association in Registered Nurses and Nursing Assistants: A Cross‐Sectional Multicenter Study
Source: J Nurs Manag. 2026 Feb 1;2026:3947093. doi: 10.1155/jonm/3947093 (PMC12862233; doi:10.1155/jonm/3947093)
Supplement: Supplementary file 1 — Supporting Information Additional supporting information can be found online in the Supporting Information section. [file JONM-2026-3947093-s001.docx]

# Appendices

Appendix A: STROBE Statement—Checklist of items that should be included in reports of cross-sectional studies

|  | Item No | Recommendation | Page No |
| --- | --- | --- | --- |
| **Title and abstract** | 1 | (*a*) Indicate the study’s design with a commonly used term in the title or the abstract | 1-2 |
|  |  | (*b*) Provide in the abstract an informative and balanced summary of what was done and what was found | 2-3 |
| Introduction | | | |
| Background/rationale | 2 | Explain the scientific background and rationale for the investigation being reported | 5-6 |
| Objectives | 3 | State specific objectives, including any prespecified hypotheses | 6 |
| Methods | | | |
| Study design | 4 | Present key elements of study design early in the paper | 7-8 |
| Setting | 5 | Describe the setting, locations, and relevant dates, including periods of recruitment, exposure, follow-up, and data collection | 7-8 |
| Participants | 6 | (*a*) Give the eligibility criteria, and the sources and methods of selection of participants | 7-8 |
| Variables | 7 | Clearly define all outcomes, exposures, predictors, potential confounders, and effect modifiers. Give diagnostic criteria, if applicable | 9-13 |
| Data sources/ measurement | 8* | For each variable of interest, give sources of data and details of methods of assessment (measurement). Describe comparability of assessment methods if there is more than one group | 9-13 |
| Bias | 9 | Describe any efforts to address potential sources of bias | 7 |
| Study size | 10 | Explain how the study size was arrived at | 7-8 |
| Quantitative variables | 11 | Explain how quantitative variables were handled in the analyses. If applicable, describe which groupings were chosen and why | 12-13 |
| Statistical methods | 12 | (*a*) Describe all statistical methods, including those used to control for confounding | 12-13 |
|  |  | (*b*) Describe any methods used to examine subgroups and interactions | 12-13 |
|  |  | (*c*) Explain how missing data were addressed | 12-13 |
|  |  | (*d*) If applicable, describe analytical methods taking account of sampling strategy | NA |
|  |  | (*e*) Describe any sensitivity analyses | NA |

| Results | | | |
| --- | --- | --- | --- |
| Participants | 13* | (a) Report numbers of individuals at each stage of study—eg numbers potentially eligible, examined for eligibility, confirmed eligible, included in the study, completing follow-up, and analysed | 14 |
|  |  | (b) Give reasons for non-participation at each stage | 21 |
|  |  | (c) Consider use of a flow diagram | NA |
| Descriptive data | 14* | (a) Give characteristics of study participants (eg demographic, clinical, social) and information on exposures and potential confounders | 14 + table 1 |
|  |  | (b) Indicate number of participants with missing data for each variable of interest | Table 1 |
| Outcome data | 15* | Report numbers of outcome events or summary measures | 14-18 + table 1-4 |
| Main results | 16 | (*a*) Give unadjusted estimates and, if applicable, confounder-adjusted estimates and their precision (eg, 95% confidence interval). Make clear which confounders were adjusted for and why they were included | 14-18 + table 1-4 |
|  |  | (*b*) Report category boundaries when continuous variables were categorized | 9-11 |
|  |  | (*c*) If relevant, consider translating estimates of relative risk into absolute risk for a meaningful time period | NA |
| Other analyses | 17 | Report other analyses done—eg analyses of subgroups and interactions, and sensitivity analyses | 12-18 |
| Discussion | | | |
| Key results | 18 | Summarise key results with reference to study objectives | 19-20 |
| Limitations | 19 | Discuss limitations of the study, taking into account sources of potential bias or imprecision. Discuss both direction and magnitude of any potential bias | 21 |
| Interpretation | 20 | Give a cautious overall interpretation of results considering objectives, limitations, multiplicity of analyses, results from similar studies, and other relevant evidence | 21 |
| Generalisability | 21 | Discuss the generalisability (external validity) of the study results | 21 |
| Other information | | | |
| Funding | 22 | Give the source of funding and the role of the funders for the present study and, if applicable, for the original study on which the present article is based | 24 |

*Give information separately for exposed and unexposed groups.

**Note:** An Explanation and Elaboration article discusses each checklist item and gives methodological background and published examples of transparent reporting. The STROBE checklist is best used in conjunction with this article (freely available on the Web sites of PLoS Medicine at http://www.plosmedicine.org/, Annals of Internal Medicine at http://www.annals.org/, and Epidemiology at http://www.epidem.com/). Information on the STROBE Initiative is available at www.strobe-statement.org.

Appendix B: survey instrument

| Work environment - PES-NWI (Lake, 2002) | |
| --- | --- |
| Subscale | **Item** |
| Foundations for quality care | There are opportunities for personal development or training for care workers. |
| Foundations for quality care | The management of the nursing home expects good quality of care. |
| Foundations for quality care | The nursing home is imbued with a clear vision for quality of care. |
| Foundations for quality care | The nursing home is imbued with a clear vision for quality of life and living. |
| Foundations for quality care | Competent nurses are employed |
| Foundations for quality care | Competent nursing assistants are employed |
| Foundations for quality care | Quality of care is actively monitored (quality indicators and/or resident surveys serve as basis for quality improvement) |
| Foundations for quality care | There is an induction programme for new care workers. |
| Foundations for quality care | The residential care life plans are up to date for all residents. |
| Participation in hospital affairs | There are sufficient possibilities for growth (career opportunities) in the nursing home. |
| Participation in hospital affairs | There are sufficient opportunities for care workers to participate in policy decisions. |
| Participation in hospital affairs | The manager is very visible and accessible to staff. |
| Participation in hospital affairs | There are sufficient opportunities for career development (development throughout the career). |
| Participation in hospital affairs | The management of the nursing home listens and responds to staff concerns. |
| Participation in hospital affairs | Care workers are involved in the internal policy of the nursing home. |
| Participation in hospital affairs | Care workers have the possibility to be part of working groups. |
| Manager ability, leadership and support | The person in charge of the ward supports the care team (nurses and nursing assistants). |
| Manager ability, leadership and support | The person in charge of the ward is a good manager and supervisor. |
| Manager ability, leadership and support | There is praise and recognition for work well done. |
| Manager ability, leadership and support | The person in charge of the ward supports the care workers in making decisions, even when it concerns a disagreement with another care profession. |

| Work environment - PES-NWI (Lake, 2002) | | |
| --- | --- | --- |
| Subscale | | **Item** |
| Staffing and resource adequacy | Good logistical support (e.g. in distributing meals, making beds) enables me to spend time with the residents. |  |
| Staffing and resource adequacy | There is sufficient time and opportunity to consult with colleagues about problems in the care of residents. |  |
| Staffing and resource adequacy | There are sufficient care workers to provide quality care |  |
| Staffing and resource adequacy | There are sufficient care workers to provide quality support for living |  |
| Staffing and resource adequacy | The staffing level of care workers is sufficient to carry out the required work. |  |
| Collegial relationships in the NH ward | Describe the quality of collaboration at your workplace with colleagues in your NH ward. |  |
| Risk of Burnout - MBI (Maslach & Jackson, 1981) | |  |
| Subscale | **Item** |  |
| Emotional exhaustion | I feel emotionally exhausted by my work. |  |
| Emotional exhaustion | I feel empty at the end of the working day. |  |
| Emotional exhaustion | I feel tired when I get up in the morning and there is another working day ahead of me. |  |
| Emotional exhaustion | Working with people all day is really hard for me. |  |
| Emotional exhaustion | I feel burnt out by my work (burnout). |  |
| Emotional exhaustion | I feel frustrated by my job. |  |
| Emotional exhaustion | I feel that I work too hard for my job. |  |
| Emotional exhaustion | Working directly with people causes me too much stress. |  |
| Emotional exhaustion | I feel at the end of my rope. |  |
| Depersonalization | I don't really care what happens to some residents. |  |
| Depersonalization | I feel residents blame me for some of their problems. |  |
| Depersonalization | I feel that I treat some residents as objects. |  |
| Depersonalization | I have become more indifferent towards people since I started this job. |  |
| Depersonalization | I worry that this job makes me emotionally harder. |  |
| Personal accomplishment | I feel very energetic. |  |
| Personal accomplishment | I deal with my residents' problems very effectively. |  |
| Personal accomplishment | I have the feeling that I influence other people's lives positively. |  |
| Personal accomplishment | I can easily create a relaxed atmosphere with my residents. |  |
| Personal accomplishment | I achieve many valuable things in this job. |  |
| Personal accomplishment | I feel cheered up after working closely with residents. |  |
| Personal accomplishment | In my work, I handle emotional problems very calmly. |  |
| Personal accomplishment | I can easily understand how my residents feel |  |
